# An epigenome-wide analysis of cord blood DNA methylation reveals sex-specific effect of exposure to bisphenol A

**DOI:** 10.1038/s41598-019-48916-5

**Published:** 2019-08-26

**Authors:** Ryu Miura, Atsuko Araki, Machiko Minatoya, Kunio Miyake, Mei-Lien Chen, Sumitaka Kobayashi, Chihiro Miyashita, Jun Yamamoto, Toru Matsumura, Mayumi Ishizuka, Takeo Kubota, Reiko Kishi

**Affiliations:** 1 Hokkaido University Center for Environmental and Health Sciences, Sapporo, Japan; 2 Department of Health Sciences, Interdisciplinary Graduate School of Medicine and Engineering, University of Yamanashi, Chuo, Japan; 3 Institute of Environmental and Occupational Health Sciences, National Yang Ming University, Taipei, Taiwan; 4 Institute of Environmental Ecology, Idea Consultants, Inc., Shizuoka, Japan; 5 Department of Environmental Veterinary Sciences, Graduate School of Veterinary Medicine, Hokkaido University, Sapporo, Japan; 6 Faculty of Child Studies, Seitoku University, Chiba, Japan

**Keywords:** Epidemiology, Genetics research

## Abstract

Exposure to bisphenol A (BPA) *in utero* is associated with adverse health outcome of the offspring. Differential DNA methylation at specific CpG sites may link BPA exposure to health impacts. We examined the association of prenatal BPA exposure with genome-wide DNA methylation changes in cord blood in 277 mother-child pairs in the Hokkaido Study on Environment and Children’s Health, using the Illumina HumanMethylation 450 BeadChip. We observed that a large portion of BPA-associated differentially methylated CpGs with *p*-value < 0.0001 was hypomethylated among all newborns (91%) and female infants (98%), as opposed to being hypermethylated (88%) among males. We found 27 and 16 CpGs with a false discovery rate (FDR) < 0.05 in the analyses for males and females, respectively. Genes annotated to FDR-corrected CpGs clustered into an interconnected genetic network among males, while they rarely exhibited any interactions in females. In contrast, none of the enrichment for gene ontology (GO) terms with FDR < 0.05 was observed for genes annotated to the male-specific CpGs with *p* < 0.0001, whereas the female-specific genes were significantly enriched for GO terms related to cell adhesion. Our epigenome-wide analysis of cord blood DNA methylation implies potential sex-specific epigenome responses to BPA exposure.

## Introduction

Exposure to endocrine-disrupting chemicals (EDCs) is associated with dysfunctions of hormone-mediated processes, such as metabolism, energy balance, thyroid and reproductive functions, immune functions, and neurodevelopment^1–4^. In particular, the prenatal and early postnatal period is highly vulnerable to EDC exposure as it is the time of developmental programming essential for organogenesis and tissue differentiation^5^.

Bisphenol A (BPA) is a chemical widely used in consumer products, including plastics, dental sealants, food containers, and thermal receipts^6^. Biomonitoring studies reported BPA was detectable in the dust, air particles, and water^7^. Humans are exposed to this compound through their diet, inhalation of house dust, and skin contact^8^. As a result, BPA is widely found in different populations, including children and pregnant women^9,10^. Furthermore, BPA is a known EDC; it has been shown to have estrogenic effects through binding to the estrogen receptors (ERs)^11^. BPA can also activate a variety of growth-related transcription factors and bind effectively to several nuclear receptors involved in cell maturation^12,13^. Additionally, BPA has agonistic and antagonistic effects on thyroid function^14^. Numerous experimental studies indicate that early-life exposure to BPA affects metabolic, reproductive, and behavioral phenotypes of the offspring and has sustained effects on future health trajectories^15–17^. Given its widespread human exposure and endocrine-disrupting effects, developmental BPA exposure could have adverse effects on human health. Epidemiological studies have shown that prenatal BPA exposures are associated with various health outcomes including altering birth size^18,19^, disruption of hormone balance^20,21^, obesity^22,23^, immune function impairment^24,25^, and neurobehavioral problems^26–32^, and most of these effects were sex-specific.

The actual mechanisms accounting for long-term effects of early-life exposure to EDCs remain unclear. Epigenetic is the study of the biological mechanism of heritable and reversible chemical modifications of chromatin that regulate gene expression without changing the DNA sequence^33^. Epigenetic alterations play a role in embryonic development and cellular differentiation^34^ and can be affected by environmental factors, primarily when this occurs within the sensitive developmental windows^35^. Accumulating evidence suggests that epigenetic alterations may link developmental EDC exposure with susceptibility to diseases later in life^36–39^. DNA methylation is among the most studied mechanisms of epigenetic regulation^40^ and may be one of the mechanisms by which BPA exposure exerts its biological effects^41^. Evidence from experimental studies suggests that DNA methylation changes in the offspring can occur in response to developmental BPA exposure^40,42–44^. Previous human cohort studies showed that prenatal BPA exposure associated with DNA methylation profiles of fetal liver genes^45–47^ and metabolism-related genes of the offspring^48,49^. Furthermore, it has been shown that BPA-induced epigenetic effects are usually sex-specific^49,50^.

Genome-wide methylation analysis allows unbiased assessment of epigenetic alterations in relation to the environmental factors^51^; however, only one cohort study from Germany showed an association between maternal urinary BPA levels (low levels with <7.6 ng/mg creatinine; *n* = 102 and high levels with >15.9 ng/mg creatinine; *n* = 101) and genome-wide DNA methylation in cord blood samples regardless of infant’s sex^52^. We aimed to examine cord blood DNA methylation changes in association with BPA exposure by an epigenome-wide association study (EWAS) in a Japanese cohort. Besides, analyses were also performed to determine sex-specific differences in BPA exposure-associated methylation profiles.

## Results

### Study characteristics

The characteristics of the participants with the corresponding median BPA concentrations in cord blood are described in Table 1. BPA was detected in 68.6% of cord blood samples. The median of cord blood BPA was 0.050 ng/mL (Interquartile range (IQR): <the limit of quantification (LOQ) – 0.075). The average ± standard deviation (s.d.) age of the mothers was 30.0 ± 4.9 years. Of the 277 newborns, 123 (44.4%) were male. None of the characteristics shown in Table 1 were significantly associated with BPA levels. The comparison of BPA levels and maternal and infant characteristics between infant sexes are shown in Supplementary Table S1. There was a significant difference in birth weight between the infant sexes. Besides, the median level of BPA, maternal characteristics, and gestational age were not significantly different between sexes. The percentage of subjects with BPA values below the LOQ among the females (32.5%) was slightly higher than that among the males (30.1%).

**Table 1:** Cord blood BPA concentrations (ng/mL) in relation to the characteristics of mothers and infants.

		N (%) or Mean ± SD	BPA (ng/mL)	
			Median (25th, 75th) or correlation (ρ)	*p*-value
**Maternal Characteristics**				
Maternal Age (year)^a^		30.0 ± 4.9	ρ = −0.031	0.613
Prenatal-BMI (kg/m^2^)^a^		20.9 ± 2.9	ρ = −0.031	0.605
Parity^b^	0	145 (52.3)	0.054 (0.020, 0.076)	0.407
	≧1	132 (47.7)	0.047 (0.020, 0.074)	
Educational level (year)^b^				
	≦12	123 (44.4)	0.050 (0.020, 0.076)	0.990
	>12	154 (55.6)	0.052 (0.020, 0.070)	
Annual household income (million yen)^c^				
	<3	51 (18.5)	0.055 (0.020, 0.072)	0.829
	3–5	144 (52.4)	0.053 (0.020, 0.076)	
	5–7	59 (21.5)	0.048 (0.020, 0.068)	
	>7	21 (7.6)	0.044 (0.020, 0.085)	
Smoking during pregnancy^b^				
	No	234 (84.5)	0.051 (0.020, 0.075)	0.941
	Yes	43 (15.5)	0.056 (0.020, 0.072)	
Alcohol consumption during pregnancy^b^				
	No	183 (66.1)	0.052 (0.020, 0.075)	0.831
	Yes	94 (33.9)	0.047 (0.020, 0.075)	
Caffeine intake during pregnancy (mg/day)^a^				
		148.7 ± 121.9	ρ = −0.012	0.843
**Infant Characteristic**				
Gestation age (week)^a^		39.8 ± 1.0	ρ = −0.003	0.964
Sex^b^	Male	123 (44.4)	0.056 (0.020, 0.075)	0.429
	Female	154 (55.6)	0.047 (0.020, 0.073)	
Birth weight (g)^a^		3131.3 ± 333.5	ρ = 0.060	0.324

^a^Spearman’s correlation test (ρ), ^b^Mann-Whitney U-test, ^c^Kruskal-Wallis test.

### Epigenome-wide association study of in utero BPA exposure

**Figure 1:**
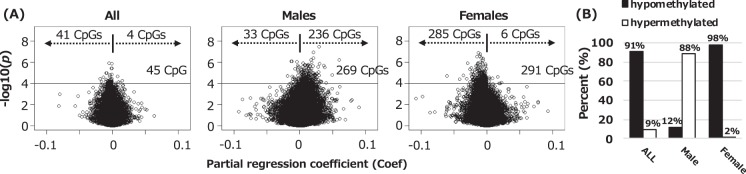
(**A**) Volcano plots of the log_10_(*p*-values) versus the magnitude of effect (Coef) for the genome-wide analysis of the association between BPA exposure and DNA methylation in cord blood among all newborns, male infants, and female infants. Horizontal lines represent a *p*-value < 0.0001. (**B**) The percentage of hypo and hypermethylated CpGs with *p* < 0.0001 found in the analyses for all, male, and female infants.

Our analysis showed that *p*-value distribution of all CpGs was generally similar to the theoretical distribution among all newborns (in the quantile-quantile plot, genomic inflation factor: λ = 1.01), whereas the *p*-values distribution deviated from the theoretical distribution among male and female infants (λ = 1.22 and λ = 1.35, respectively) as shown in Supplementary Fig. S1. Volcano plots of EWAS analyses for all newborns, male infants, and female infants showed an imbalance in positive versus negative methylation changes (Fig. 1A), suggesting a global methylation shift. As we had too few false discovery rate (FDR)-significant findings to confirm the sex-specific effect on DNA methylation changes, we compared CpGs with uncorrected *p*-value < 0.0001 (45 CpGs in all newborns, 269 CpGs in male infants, and 291 CpGs in female infants) and observed a large portion of these was hypomethylated among all newborns (91%) and female infants (98%) (Fig. 1B). In contrast, of the 269 CpGs among male infants, 236 CpGs (88%) were hypermethylated (Fig. 1B). All CpGs with *p*-value < 0.0001 are listed in Supplementary Tables S2–S4. Of those, two CpGs; cg25857471 (*DPCR1*) and cg13481969 (*LOC441455*), were overlapping between all newborns and males, and nine CpGs; cg08710564 (*ST5*), cg23279887 (*TMEM161A*), cg11820931 (*DDX21*), cg23047671 (*LINC01019*), cg14048686 (*PPP1R26-AS1*), cg27624753 (*CRAMP1L*), cg02344993 (*CLTC*), cg27038101 (*CTRL*), cg12393623 (*METRNL*), were overlapping between all newborns and females (please refer to the Supplementary Fig. S2 and Table S2). There were no overlaps between males and females.

**Figure 2:**
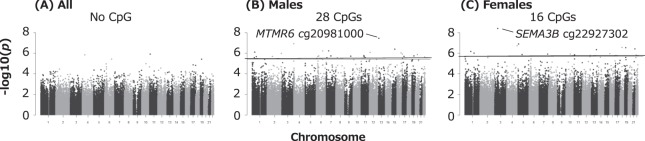
Manhattan plots of *p*-value associations between BPA exposure and DNA methylation across chromosomes in analyses for (**A**) all newborns, (**B**) male infants, and (**C**) female infants. Horizontal lines represent the significance threshold of FDR q < 0.05.

Next, we studied differentially methylated probes (DMPs) with epigenome-wide significant methylation changes; an FDR *q*-value < 0.05. As shown in Manhattan plots of genome-wide analyses (Fig. 2), we observed 28 male- and 16 female-specific DMPs that are listed in Tables 2 and 3, respectively. In the male-only analysis, BPA levels in cord blood were associated with hypermethylation of 22 DMPs and hypomethylation of 6 DMPs. Among the female infants, BPA levels were associated with hypomethylation of 16 DMPs. There were no CpGs with FDR < 0.05 in all newborns (Fig. 2A); however, the directions of methylation changes at DMPs observed in all infants were consistent with those found in the sex-stratified analyses (see Tables 2 and 3).

**Table 2:** 28 CpGs with DNA methylation levels in cord blood associated with BPA concentrations with FDR q < 0.05 in analysis for male infants.

CpG	Chr	Position	Gene	Relation to gens	Relation to island	Sapporo				Taiwan^a^		Replicated for the direction
						Male infants^b^		All newborns^c^		All newborns		
						Coef^d^	*p*-value	Coef^d^	*p*-value	Coef^d^	*p*-value	
cg25275331	1	40723935	*ZMPSTE24*	1stExon	island	−0.013	7.91E-07	−0.003	0.100	0.003	0.215	
cg21115004	1	67520352	*SLC35D1*	TSS1500	shore	0.015	2.15E-06	0.002	0.241	0.002	0.587	✓
cg02920421	1	32572534	*KPNA6*	TSS1500	shore	0.010	2.70E-06	0.004	0.034	0.001	0.591	✓
cg04627863	2	234741670	*HJURP*	IGR	open sea	0.018	1.01E-06	0.009	0.001	0.000	0.966	✓
cg08527179	2	85132050	*TMSB10*	TSS1500	island	0.012	1.98E-06	0.004	0.005	−0.001	0.646	
cg23605991	4	618114	*PDE6B*	TSS1500	shore	0.021	1.19E-07	0.005	0.085	0.001	0.635	✓
cg01119278	6	110721349	*DDO*	Body	island	0.060	5.83E-07	0.017	0.094	−0.006	0.581	
cg00050375	6	43404828	*ABCC10*	Body	open sea	0.009	1.78E-06	0.002	0.189	−0.003	0.254	
cg20620326	6	28715664	*LOC401242*	IGR	open sea	0.020	2.20E-06	0.003	0.487	−0.002	0.555	
cg02330394	6	10695063	*C6orf52*	TSS1500	island	−0.003	3.19E-06	−0.001	0.050	0.000	0.718	
cg22674202	7	37531818	*GPR141*	IGR	open sea	0.017	5.97E-07	0.005	0.079	−0.005	0.130	
cg17833862	7	73790277	*CLIP2*	Body	island	0.007	1.85E-06	0.003	0.032	−0.001	0.681	
cg00507727	7	8302191	*ICA1*	TSS200	island	−0.002	2.65E-06	−0.001	0.002	0.001	0.263	
cg14253670	8	24802149	*NEFL*	IGR	shelf	0.028	2.63E-06	0.010	0.008	−0.004	0.204	
cg13481969	9	99449345	*LOC441455*	IGR	island	−0.003	1.94E-06	−0.002	6.28E-05	0.001	0.635	
cg03470671	11	45115584	*PRDM11*	1stExon	open sea	0.012	1.71E-06	0.004	0.021	−0.004	0.173	
cg07637188	11	47346161	*MADD*	Body	open sea	0.011	2.19E-06	0.004	0.014	−0.000	0.870	
cg03687707	11	63745967	*COX8A*	IGR	shelf	0.004	2.68E-06	0.002	0.022	−0.002	0.074	
cg21372595	12	14136152	*ATF7IP*	IGR	shore	−0.012	8.53E-07	−0.005	0.013	0.000	0.901	
cg20981000	13	25791388	*MTMR6*	IGR	open sea	0.030	3.44E-08	0.004	0.313	0.000	0.991	✓
cg20796298	15	65687852	*IGDCC4*	Body	shore	0.021	3.97E-07	0.006	0.069	−0.000	0.943	
cg00120998	16	67881473	*CENPT*	TSS200	island	−0.005	9.42E-07	−0.002	0.023	0.001	0.466	
cg07130392	16	89588927	*SPG7*	Body	island	0.005	1.20E-06	0.001	0.187	0.000	0.661	✓
cg23798387	17	26972805	*KIAA0100*	TSS1500	shore	0.036	1.99E-06	0.008	0.203	0.003	0.703	✓
cg06126721	17	1478065	*SLC43A2*	3′UTR	island	0.038	3.01E-06	0.007	0.234	−0.006	0.369	
cg17922329	19	35646039	*FXYD5*	5′UTR	island	0.007	1.77E-06	0.002	0.077	0.003	0.147	✓
cg13636640	20	31349939	*DNMT3B*	TSS1500	shore	0.030	1.38E-06	0.011	0.010	NA	NA	
cg19353578	22	39634386	*PDGFB*	Body	shelf	0.016	2.23E-06	0.003	0.141	0.001	0.791	✓

^a^Linear regression analyses adjusted for the sex of child were applied to determine the associations of DNA methylation levels with *ln*-transformed BPA levels in the Taiwan cohort.

^b^Adjusted for maternal age, maternal educational levels, maternal pre-pregnancy BMI, maternal smoking during pregnancy, gestational age, and cord blood cell estimates.

^c^Adjusted for maternal age, maternal educational levels, maternal pre-pregnancy BMI, maternal smoking during pregnancy, gestational age, infant sex, and cord blood cell estimates.

^d^Partial regression coefficient indicates absolute DNA methylation change per ln-unit increase in BPA concentration.

Chr, chromosome; TSS, transcription start site; TSS200, 200 bases from TSS; TSS1500, 1500 bases from TSS; Body; gene body; UTR, untranslated region; NA, not available.

**Table 3:** 16 CpGs with DNA methylation levels in cord blood associated with BPA concentrations with FDR *q < *0.05 in analysis for female infants.

CpG	Chr	Position	Gene	Relation to gens	Relation to island	Sapporo				Taiwan^a^		Replicated for the direction
						Female infants^b^		All newborns^c^		All newborns		
						Coef^d^	*p*-value	Coef^d^	*p*-value	Coef^d^	*p*-value	
cg12061021	1	95699574	*RWDD3*	TSS200	shore	−0.006	6.55E-07	−0.004	0.000	−0.001	0.732	✓
cg21461470	1	149821425	*HIST2H2AA4*	TSS1500	shore	−0.010	1.01E-06	−0.006	0.001	0.000	0.948	
cg22927302	3	50304463	*SEMA3B*	TSS1500	open sea	−0.015	4.00E-09	−0.005	0.014	NA	NA	
cg23603782	4	173884384	*GALNTL6*	Body	open sea	−0.010	1.81E-07	−0.006	0.000	−0.001	0.639	✓
cg27629673	5	7462856	*ADCY2*	Body	open sea	−0.013	1.27E-07	−0.005	0.029	−0.000	0.905	✓
cg22465281	5	65017296	*NLN*	TSS1500	shore	−0.014	1.36E-06	−0.005	0.036	NA	NA	
cg19734222	7	142020183	*PRSS58*	IGR	open sea	−0.030	1.25E-06	−0.011	0.030	0.002	0.734	
cg11820931	10	70717302	*DDX21*	Body	shore	−0.013	1.23E-06	−0.009	1.00E-05	−0.005	0.354	✓
cg08710564	11	8829438	*ST5*	5′UTR	open sea	−0.007	1.45E-06	−0.005	1.24E-06	−0.001	0.363	✓
cg17339927	12	132991124	*FBRSL1*	IGR	shore	−0.014	1.63E-06	−0.007	0.006	−0.004	0.249	✓
cg15193473	13	103453215	*BIVM*	5′UTR	island	−0.006	4.53E-07	−0.003	0.000	0.001	0.467	
cg27624753	16	1673702	*CRAMP1L*	Body	shelf	−0.007	1.10E-06	−0.005	6.65E-05	−0.004	0.097	✓
cg27638035	18	35015753	*BRUNOL4*	Body	open sea	−0.011	2.68E-07	−0.004	0.049	−0.002	0.341	✓
cg03636183	19	17000585	*F2RL3*	Body	shore	−0.016	2.81E-07	−0.005	0.067	−0.003	0.385	✓
cg07964219	21	46847898	*COL18A1*	Body	shore	−0.009	3.90E-07	−0.005	0.008	0.000	0.990	
cg15477600	22	21996253	*SDF2L1*	TSS1500	island	−0.002	1.57E-06	−0.001	0.004	−0.000	0.691	✓

^a^Linear regression analyses adjusted for the sex of child were applied to determine the associations of DNA methylation levels with ln-transformed BPA levels in the Taiwan cohort.

^b^Adjusted for maternal age, maternal educational levels, maternal pre-pregnancy BMI, maternal smoking during pregnancy, gestational age, and cord blood cell estimates.

^c^Adjusted for maternal age, maternal educational levels, maternal pre-pregnancy BMI, maternal smoking during pregnancy, gestational age, infant sex, and cord blood cell estimates.

^d^Partial regression coefficient indicates absolute DNA methylation change per ln-unit increase in BPA concentration.

Chr, chromosome; TSS, transcription start site; TSS200, 200 bases from TSS; TSS1500, 1500 bases from TSS; Body; gene body; UTR, untranslated region; NA, not available.

We explored whether FDR-significant DMPs identified in the Sapporo cohort also showed the same direction of methylation change in a Taiwan cohort (the right columns in Tables 2 and 3). Of the DMPs, one male- and two female-specific DMPs were not available for the analysis. Ten of the fourteen female-specific DMPs showed the same direction of methylation changes (hypomethylation) in both cohorts, whereas the direction of methylation changes in the male-specific DMPs was not reproducible (9 of 27 male-specific DMPs).

Sensitivity analyses showed that neither maternal smoking nor subjects with BPA levels below LOQ were associated with male-specific hypermethylation and female-specific hypomethylation, as shown in Supplementary Fig. S3. The percentage of hypermethylated CpGs among males in the analyses for maternal smoking and BPA levels below the LOQ were 82% and 92%, respectively. Among females, 94% of CpGs for maternal smoking and 87% of CpGs for BPA levels below were hypomethylated. The analysis for the association between methylation and tertile BPA levels, which were <0.041, 0.041–0.066, and >0.066 ng/mL, also showed predominant hypermethylation (94%) among males and hypomethylation (93%) among females (Supplementary Fig. S3) as with the analyses for *ln*-transformed BPA levels. CpGs with p-value < 0.0001 in the analyses for tertile BPA levels are listed in the Supplementary Tables S5–S7. Twenty-seven of the 28 male-specific DMPs and all sixteen female-specific DMPs still showed a p-value < 0.0001 in the sex-stratified analyses for tertile BPA (Supplementary Tables S6 and S7). It is noted that those additional analyses again showed high inflation factors (from 1.2 to 1.4). We have also examined associations between the predicted cord blood cell proportions from the 450 K array and BPA levels among all infants, infants with <LOQ, and >LOQ and found no significant correlation between them (Supplementary Table S8).

Furthermore, we also investigated two CpGs in *MEST* and *RAB40B* identified by a previous EWAS analysis on prenatal BPA exposure and cord blood DNA methylation^52^ based on our dataset among all newborns. The CpG in *MEST* displayed the same direction of methylation change, although it did not reach statistical significance. On the contrary, the trend was not replicated at CpG in *RAB40B*. Meanwhile, an animal model showed that the effect of prenatal exposure to BPA on the brain was mediated by X-chromosome inactivation^53^. An enrichment of hypomethylated sites on the X-chromosome due to increasing BPA concentrations was observed in young girls^54^. It is possible that prenatal BPA exposure has effects on DNA methylation alterations at CpGs on X-chromosome. To examine the possibility, we performed sex-stratified analyses, including CpGs on the X-chromosome; however, we observed no enrichment of differentially methylated CpGs on X-chromosome.

### Network analysis of DMPs

**Figure 3:**
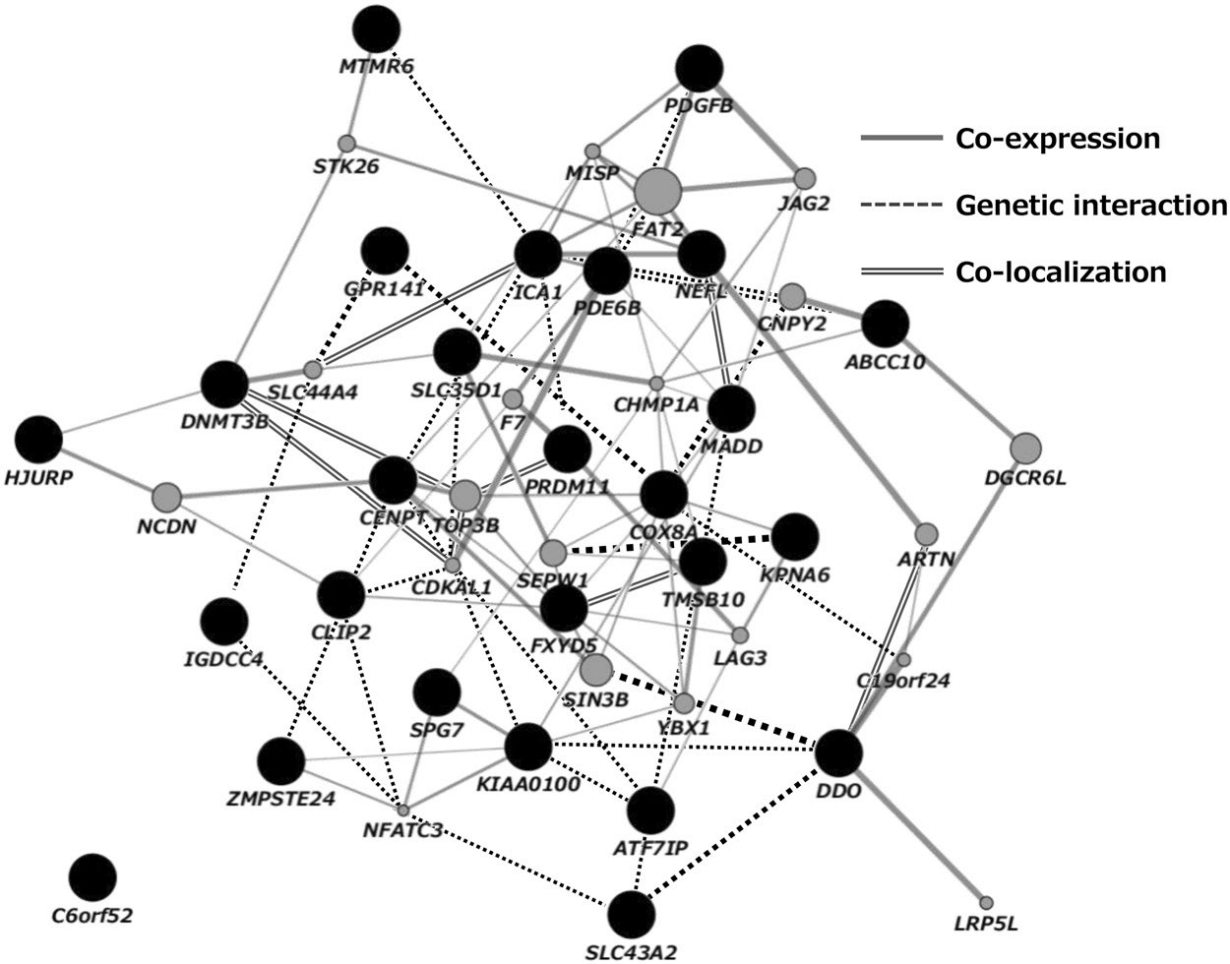
Gene network analysis using GeneMANIA. Dark circles represent genes associated with the 26 DMPs found to be related to BPA exposure in the male-only analysis. Light circles represent additional genes predicted by GeneMANIA based on genetic and physical interactions.

**Figure 4:**
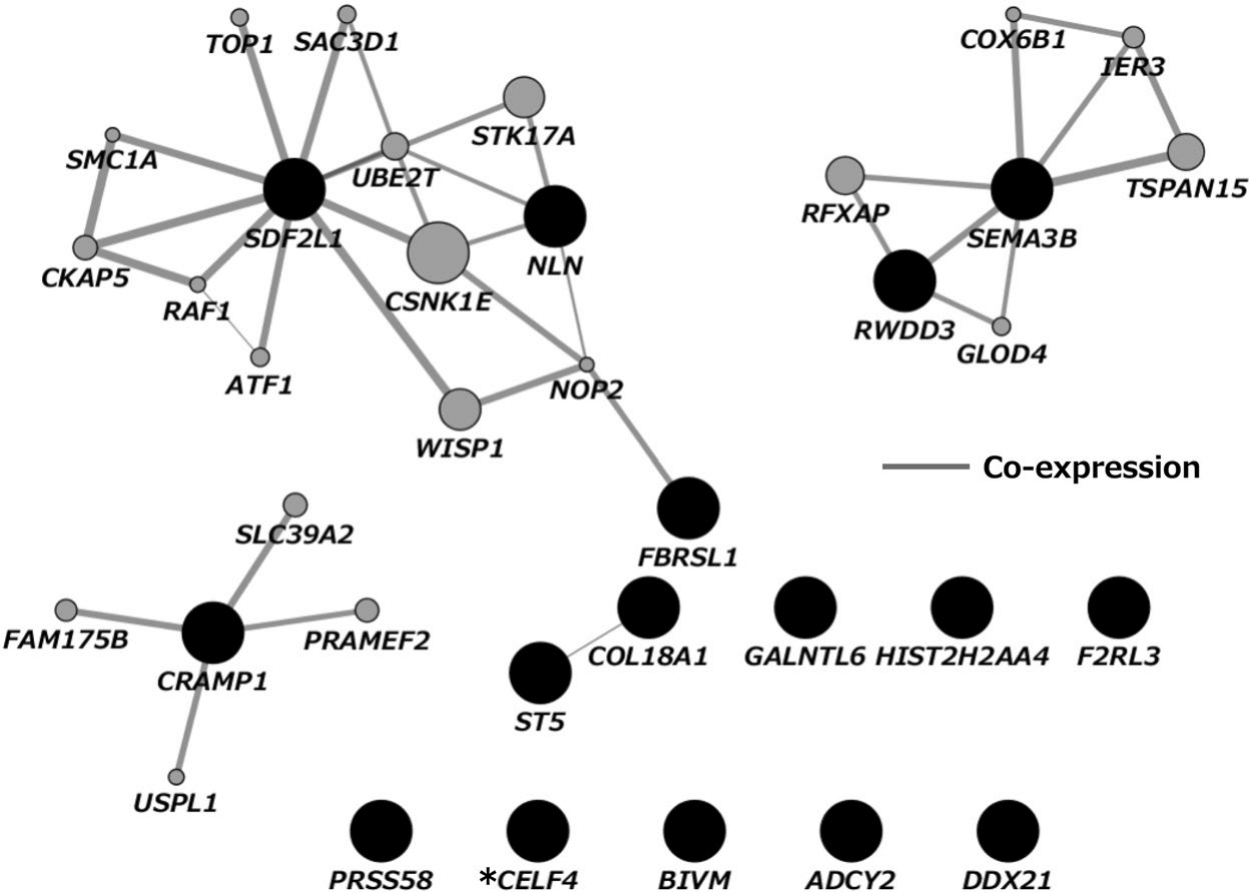
Gene network analysis using GeneMANIA. Dark circles represent genes associated with the 16 DMPs found to be related to BPA exposure in the female-only analysis. Light circles represent additional genes predicted by GeneMANIA based on genetic and physical interactions. *CELF4, an alias for *BRUNOL4*.

Genes annotated to DMPs (28 male-specific and 16 female-specific genes) were analyzed by network analysis using GeneMANIA^55^ (https://genemania.org/) that is based on known genetic and physical interactions, shared pathways and protein domains as well as protein co-expression data. Two male-specific genes (*LOC401242* and *LOC441455*) were not available for GeneMANIA analysis; therefore, only 26 of the 28 male-specific genes were included in this analysis. The analyses showed that all male-specific genes, except one (*C6orf52*), formed a compact cluster showing co-expression, genetic interaction, and colocalization (see Fig. 3). None of the networks of pathways, physical infarctions, predicted, or shared protein domains were extracted by GeneMANIA. Conversely, among the 16 female-specific genes, six genes showed three disperse clusters of co-expression (Fig. 4). The rest of the genes showed no interaction with others.

### Gene ontology analysis

We also investigated the underlying biology that may be affected by BPA-associated variations in a sex-specific manner. As we were not able to perform enrichment analysis on only 28 DMPs in males and 16 DMPs in females, we tested for gene ontology (GO) terms and Kyoto Encyclopedia Genes and Genomes (KEGG) pathways^56^ enrichment among the CpGs associated with BPA levels with *p* < 0.0001. Four GO terms among the female infants were significant at FDR < 0.05, as shown in Table 4. None of the GO terms among the males were substantial at the FDR threshold. In contrast, the gene set for both sexes were enriched with genes from numerous KEGG pathways. The top ten enriched pathways ranked by the lowest *p*-value, excluding the pathways for diseases, are shown in Table 5. Among males, six of the ten pathways are involved in signal transduction – MAPK signaling pathway, AMPK signaling pathway, Rap1 signaling pathway, Signaling pathways regulating pluripotency of stem cells, mTOR signaling pathway, and Phospholipase D signaling pathway. Among the ten pathways enriched among the female, three pathways were associated with the endocrine system – estrogen signaling pathway, relaxin signaling pathway, and parathyroid hormone synthesis, secretion, and action.

**Table 4:** Significantly enriched GO terms (FDR < 0.05) for the CpGs with *p*-value < 0.0001 from the female-only analysis.

GO term	Ontology	N	DE	*p*-value	FDR
homophilic cell adhesion via plasma membrane adhesion molecules	BP	149	22	4.57E-12	9.46E-08
cell-cell adhesion via plasma-membrane adhesion molecules	BP	210	22	5.28E-10	5.46E-06
cell-cell adhesion	BP	845	38	1.92E-07	0.001
calcium ion binding	MF	652	30	2.17E-06	0.011

N, number of genes represented by a GO term; DE, number of genes from the GO term that were included in the CpGs with *p* < 0.0001; FDR, FDR-adjusted *p*-value for enrichment; BP, biological process; MF, molecular function.

**Table 5:** Top 10 enriched KEGG pathways ranked by the lowest *p*-value associated with genes annotated to the CpGs with *p < *0.0001 from the male- and female-only analyses.

KEGG pathway description	N	DE	P.DE	FDR
**Male only analysis**				
Phagosome	142	5	3.85E-05	0.002
MAPK signaling pathway	283	7	6.28E-05	0.002
AMPK signaling pathway	117	5	7.51E-05	0.002
Focal adhesion	191	6	8.16E-05	0.002
Rap1 signaling pathway	203	6	8.60E-05	0.002
Signaling pathways regulating pluripotency of stem cells	135	5	1.35E-04	0.003
mTOR signaling pathway	147	5	1.59E-04	0.003
Cellular senescence	153	5	1.72E-04	0.003
Phospholipase D signaling pathway	142	5	2.07E-04	0.003
Natural killer cell-mediated cytotoxicity	105	4	2.29E-04	0.003
**Female only analysis**				
cAMP signaling pathway	192	8	9.13E-07	1.5E-04
Estrogen signaling pathway	136	7	9.36E-07	1.5E-04
Relaxin signaling pathway	125	6	1.19E-05	0.001
Th17 cell differentiation	102	5	3.43E-05	0.001
Wnt signaling pathway	140	6	3.54E-05	0.001
Cell cycle	121	5	7.76E-05	0.002
Platelet activation	122	5	9.80E-05	0.002
B cell receptor signaling pathway	68	4	1.20E-04	0.003
Parathyroid hormone synthesis, secretion, and action	105	5	1.21E-04	0.003
Cytokine-cytokine receptor interaction	265	5	2.64E-04	0.004

N, total genes in the KEGG pathway; DE, the number of genes within the CpGs with *p* < 0.0001; FDR, FDR-adjusted p-value for enrichment.

## Discussion

Even though BPA levels in this study were relatively lower^20^ than those in the previous studies on cord blood BPA levels^19,57,58^, we found substantial sex differences in methylation changes associated with BPA exposure. Among males, BPA exposure was more frequently associated with hypermethylation than with hypomethylation, whereas it was associated predominantly with hypomethylation among female infants. Genes annotated to these CpGs also showed sex differences in the genetic network and functional enrichment analyses. Our results suggest that even at low levels, BPA exposure may impact DNA methylation status at birth in a sex-specific manner.

Among males, the top hit showing hypermethylation, cg20981000, is located in the intergenic region (*IGR*) of *MTMR6*, which encodes myotubularin related protein 6 (Fig. 2 and Table 2). Notably, based on Comparative Toxicogenomic Database (CTD, hhtp://ctdbase.org//) which provides manually curated information on environmental chemicals, interacting genes, and associated health effects in human and animal models, experimental models have reported that exposure to BPA resulted in decreased expression of *MTMR6* mRNA^59^ and increased DNA methylation of *MTMR6* gene^60^. In the female-only analysis, the most significant DMP (cg22927302) was mapped to *SEMA3B*, which encodes Semaphorin 3B (Fig. 2 and Table 3). BPA exposure also affected the expression of *SEMA3B* mRNA^61,62^ and decreased DNA methylation of *SEMA3B* promoter^63^ in experimental models. In addition, according to CTD, among nine genes annotated to DMPs with p-value < 0.0001 overlapping between all newborns and females, methylation and/or expression of seven genes: *ST5* (Suppression of Tumorigenicity 5)^61,64,65^, *TMEM161A* (Transmembrane Protein 161A)^61,66^, *DDX21* (DExD-Box Helicase 21)^60,61,66,67^, *CRAMP1L* (Cramped Chromatin Regulator Homolog 1)^59,61^, *CLTC* (Clathrin Heavy Chain)^61^, *CTRL* (Chymotrypsin Like)^61,65,66^, and *METRNL* (Meteorin Like, Glial Cell Differentiation Regulator)^61^ were disrupted by *in vivo/vitro* BPA exposure. Meanwhile, the magnitude of the effect on DNA methylation change represented in the absolute value of the regression coefficient (Coef) at the DMPs was generally small (see Tables 2 and 3). Since DNA methylation is tissue-specific, a small difference in methylation levels may result from a small fraction of cells exhibiting the difference at a particular CpG. Among FDR-corrected DMPs, only one male-specific DMP (cg01119278) in the gene body of *DDO* (D-Aspartate Oxidase) showed a positive association with the value of Coef >0.05 (Table 2). According to CTD, an *in vitro* study has shown that exposure to BPA decreased mRNA level of *DDO*^61^. We also found a DMP mapped to *DNMT3B* in the analysis for male infants (Table 2). Gestational BPA exposure can alter the expression of DNA methyltransferases (DNMTs) in the mouse brain, altering methylation and expression of ERα^41^. This is likely one of the mechanisms by which BPA exerts its endocrine-disrupting effects^16^. However, there might be other epigenetic mechanisms, such as histone modification^68,69^ or expression non-cording RNAs^70–74^, underlying prenatal BPA exposure. Further studies are needed to investigate this issue.

Despite no differences in BPA levels between the sexes (Supplementary Table S1), methylation changes derived from exposures to BPA showed notable sex-specific differences – the inversed direction of effects on methylation changes might be responsible for the lack of significant changes among all newborns. The observed preference of BPA-induced hypomethylation in females, as opposed to hypermethylation among males, is consistent with the findings from studies on blood-based methylation alterations that showed BPA-induced hypomethylation in women^75^ and young girls^54^. However, we observed no enrichment of hypomethylated CpGs on X-chromosome as noted in young girls^54^. In the gene-specific analyses, Montrose *et al*.^49^ showed that prenatal BPA exposure was associated with a decrease in cord blood *IGF2* and *PPARA* methylation among females. BPA is considered to predominantly induce hypomethylation in females, while its effects in males are unclear^76^.

Replication analyses using a different population is vital to validate the result from epigenome-wide analyses. It is desirable that the platform, sample matrices, statistical model, and ancestry are aligned in both cohorts. As far as we knew, among Asian cohorts, only the Taiwan cohort had both maternal BPA concentration and the 450 K methylation data in cord blood. In the Taiwan cohort, the concentrations of BPA in maternal urine samples during the third trimester were measured^77^. According to Montrose *et al*.^49^, the geometric mean and standard deviation for BPA concentrations measured in urine and plasma were similar; however, the distribution range of BPA levels in the maternal urine in the Taiwan cohort (0.03 to 125.75 μg/g creatine) was broader compared to the distribution of cord blood BPA levels in the Sapporo cohort (LOQ; 0.04 ng/mL to 0.22 ng/mL). Despite the differences in sample matrices and BPA measuring timing, ten of the 14 female-specific DMPs (71%) had the same direction of methylation change (hypomethylation) in both Sapporo and Taiwanese cohorts. Whereas, only nine of the 27 male-specific DMPs showed the same direction of methylation change in both cohorts (Tables 2 and 3). Although we should notice that the concentrations of BPA were determined using different biological specimens, it is possible that the variation in BPA-associated effect spectrum over BPA levels^78^ may explain the very limited agreement in the males between these two cohorts. The difference in the time of BPA measurement and/or shorter gestational age may also account for the disparities. As mentioned above, the effects of BPA on DNA methylation in male infants has not been clarified^76^. Further studies would be required to evaluate its effects among males.

Sex-stratified analyses showed inflation of the estimates, as shown in Supplementary Fig. S1. We applied the ComBat method to reduce batch effects and adjusted for cord cell proportion estimates to remove issues associated with cell heterogeneity. Given that there was no inflation among all newborns, the inflation in sex-stratified analysis cannot just reflect strong signals for some phenotypes (e.g., gestational age, birth weight, and maternal smoking). For instance, the sensitivity analyses showed that the observed sex-specific associations between BPA exposure and methylation alterations were mostly independent of maternal smoking during pregnancy that was most likely a residual confounder^79,80^. Sample size reduced by half in sex-stratified analyses might cause significant deviation from the expected distribution of p-value. Nevertheless, two randomly divided groups did not show inflation (data not shown). Another factor that might be driving a considerable proportion of significant results was a massive tail of subjects with BPA levels below LOQ. However, both analyses excluding subjects with BPA levels below the LOQ and for tertiles of BPA levels again showed the inflated results with male-specific hypermethylation and female-specific hypomethylation (Supplementary Fig. S2). Although there remains a possibility that population substructure in sex-stratified groups may bias or influence the results, it is assumed that BPA exposure might affect genome-wide methylation levels with a sex-specific association. Whereas, over inflation might lead to false discoveries in our analyses. We need to replicate our findings by further investigations with larger sample sizes and comparable exposure ranges.

In addition to the opposed direction of effect between sexes, the network analyses of genes annotated to FDR-corrected DMPs showed the sex-difference (Figs 3 and 4). The male-specific genes clustered into a complex interconnected network, whereas the female-specific genes were isolated. In contrast, with regard to the gene pathways, the female-specific CpGs with *p*-value < 0.0001 included genes that were significantly enriched for GO terms related to cell adhesion (FDR < 0.05) – homophilic cell adhesion via plasma membrane adhesion molecules, cell-cell adhesion via plasma membrane adhesion molecules, cell-cell adhesion, and calcium ion binding (Table 4). On the contrary, genes annotated to the male-specific probes were not enriched in any GO terms with an FDR threshold. With respect to KEGG pathways, it should be noted that enrichment in the MAPK signaling pathway and the estrogen signaling pathway were observed among the genes annotated to male- and female-specific probes, respectively. According to Singh and Li^81^, a mitogen-activated protein (MAPK) and estrogen receptor (ESR) were included in most frequently curated BPA-interacting genes/proteins. Nevertheless, it is possible that the observed differences between infant sex could relate to cord blood cell proportions. We compared the cell type proportions between sex and found a significant difference in the CD8T cell type (Supplementary Table S9). There remains a possibility that different cell compositions influence the observed distinct results between infant sexes.

A previous study on epigenetic effects of prenatal BPA exposure using the genome-wide analysis reported cord blood DNA methylation differences at two CpGs in *MEST* and *RAB40B* between subjects with low (*n* = 102, <7.6 ng/mg creatinine) and high (*n* = 101, >15.9 ng/mg creatinine) maternal urinary BPA levels at 34 weeks of pregnancy in Leipzig, Germany^52^. Since the analysis was not stratified by sex, to be precise, we investigated the methylation changes at the CpGs on *MEST* and *RAB40B* based on our dataset among all newborns. The CpG in *MEST* displayed the same direction of methylation change as those reported by Junge *et al*.^52^, although it did not reach statistical significance. On the contrary, the trend was not replicated at CpG in *RAB40B*. It is plausible that differences in sample matrices, statistical model, and ancestry might be partially responsible for the disagreement. We need more research focused on sex-specific associations to elucidate the potential role of BPA in methylation alteration.

In previous studies using the same Sapporo cohort, we had reported that BPA exposure is associated with reproductive hormone levels of neonates^20^, child behavioral problems at an early age^28^, and fetal metabolic-related biomarkers^82^ in a sex-specific manner. Other epidemiological studies also showed that the impact of developmental BPA exposure on neurobehavioral functions might differ between the sexes^83,84^. Harley *et al*.^22^ showed that increasing BPA concentrations in mothers during pregnancy was associated with decreased body mass index (BMI), body fat, and overweight/obesity at nine years of age in girls but not boys. Sex-specific methylomic profiles may underlie the differences in gene expression and functions^85^ and contribute to a differential susceptibility between males and females to adverse health outcomes as seen in animal models, which were used to evaluated life-course sexually dimorphic health effects following developmental BPA expouure^50,68,86–88^. Cell adhesion molecules are utilized in various steps during embryonic development and cellular differentiation. Possibly, female-specific hypomethylation on genes related to cell adhesion may increase the gene expression and mediate metabolic outcomes, including changes in body weight and body fat. The effect of methylation changes identified herein on sex-specific health outcomes needs to be elucidated in a later stage. Meanwhile, our results corroborate the need for studies on the sex-specific effects of EDCs on DNA methylation alterations.

The following limitations of this study should be considered. First, this is a cross-sectional study, and we did not determine the cause and effect relationship. Further, there have been concerns about using a single measurement of the cord blood sample as a representation of the long-term prenatal exposure due to the short half-life of BPA. Furthermore, we evaluated BPA levels in maternal urine samples during the third trimester for the replication analysis. It has been reported that BPA concentrations measured in urine and plasma are correlated^49^; however, little is known about the correlation between BPA levels with sampled at different times. Further studies should be conducted to evaluate whether one-time exposure measurement would reflect BPA exposure during pregnancy. Second, DNA methylation was measured using unfractionated cord blood. BPA is known to affect multiple tissues. Whether the associations observed in this study may reflect associations between prenatal BPA exposure and the methylation at target tissues is unknown. Third, we included participants for whom cord blood samples were available, thus limiting the scope only to mothers who delivered vaginally. It is thus possible that relatively healthier children were included in our analysis, and we may have underestimated the effects of BPA exposure. Fourth, the exposure levels of BPA were relatively low. It is possible that our results may not be generalized to the population with high exposure levels. Fifth, we analyzed CpGs showing a p-value < 0.0001, not epigenome-wide significance, to confirm the sex-specific effect on DNA methylation. That might give rise to false discoveries in our analyses. Lastly, there was a limited statistical power of our sample size for the analysis stratified by sex. Additionally, we could not perform the stratified analysis with sex for the Taiwanese cohort because of the small sample size. Therefore, we recommend further studies with larger sample sizes and comparable exposure levels.

In conclusion, this epigenome-wide study suggested that even relatively low levels of exposure to BPA impact DNA methylation status at birth in a sex-specific manner. There may be a potential susceptibility difference in relation to BPA exposure between males and females. Further studies are needed to confirm our findings and to investigate their relevance to sex-specific adverse health outcomes.

## Methods

### Study population

Participants were enrolled in the Sapporo cohort of the Hokkaido Study on Environment and Children’s Health^89–91^. Briefly, we recruited pregnant women at 23–35 weeks of gestation between 2002 and 2005 from the Toho Hospital (Sapporo, Japan). After the second trimester during their pregnancy, the participants completed the self-administered questionnaire containing baseline information including family income, educational level, parity history, and pregnancy health information including smoking status, alcohol consumption, and caffeine intake. Information on pregnancy complications, gestational age, infant sex, and birth size was obtained from medical records.

### Measurement of bisphenol A

Whole cord blood was collected immediately after birth and stored at −80 °C prior to analysis. BPA levels were measured in cord blood by using isotope dilution liquid chromatography-tandem mass spectrometry (ID-LC/MS/MS) at IDEA Consultants, Inc. (Shizuoka, Japan) as described previously^10^. The LOQ of BPA was 0.04 ng/mL.

### 450 K DNA methylation analysis

Cord blood DNA methylation at 485,577 CpGs was quantified using the Infinium HumanMethylation 450 BeadChip (Illumina Inc., San Diego, CA, USA) by G&G Science Co., Ltd. (Matsukawa, Fukushima, Japan). Details for the 450 K methylation analysis are described elsewhere^92^. Samples were run across five plate batches and were assigned randomized location across plates. After quality control^93^, functional normalization^94^ was applied to the raw data and normalized beta (*β*) values, ranging from 0–1 for 0% to 100% methylated, were obtained for the 292 cord blood samples. Probes with a detection *p*-value > 0.05 in more than 25% of samples, single nucleotide polymorphism (SNP)-affected probes, cross-reactive probes identified by Chen *et al*.^95^, and probes on sex chromosomes were removed. As a result, 426,413 CpG probes were included in the working set. We applied the ComBat method to adjust methylation data for the sample plate to reduce potential bias due to batch effects^96^. Combat-transformed *M*-values (logit-transformed *β-*values) were back-transformed to *β-values* that were used for subsequent data analyses.

### Data analyses

Among the 514 participants of the Sapporo Cohort Study, 277 mother-infant pairs had both exposure and DNA methylation data. Supplementary Fig. S4 shows the distribution of BPA levels in 277 cord blood samples. For the 87 samples below the LOQ (0.04 ng/mL), we assigned a value of half the detection limit (0.02 ng/mL). Cord blood cell proportion was estimated by the method implemented in the R/Bioconductor package *minfi*^97^. Using *limma* package in R, robust linear regression analysis^98^ and empirical Bayesian method^99^ were applied to determine the associations of β-value at each CpG site with BPA natural log (ln)-transformed concentrations, adjusted for maternal age, educational levels, pre-pregnancy BMI, smoking during pregnancy, gestational age, infant sex, and cord blood cell estimates for CD4^+^ T cells, CD8^+^ T cells, granulocytes, monocytes, B cell, and nucleated red blood cells. Adjustment covariates were selected from factors previously reported to be associated with exposure or cord blood DNA methylation. For multiple comparisons, *p*-values were adjusted by the FDR to obtain *q*-values. We further stratified the analysis by infant sex and compared CpGs with *p*-value < 0.0001 to confirm the sex-specific effect on DNA methylation changes. To address the potential residual confounding due to smoking^79,80^, we performed a sensitivity analysis, excluding infants with sustained maternal smoking during pregnancy (n = 43). Since 31.4% of BPA levels fell below the LOQ, we have also performed a sensitivity analysis excluding subjects with BPA values below LOQ (n = 87). Besides, we examined associations of tertiles of BPA levels with DNA methylation. Tertiles were <0.041, 0.041–0.066, and >0.066 ng/mL, respectively. Statistical analyses were performed using *minfi*, *sva*, and *limma* packages in R ver. 3.3.2 and Bioconductor ver. 3.3.

To further analyze underlying genetic networks of BPA-associated CpGs, we imported and analyzed genes related to DMPs surviving an FDR < 0.05 using GeneMANIA^55^ (https://genemania.org/) bioinformatics software with default parameters. We also assessed the differentially methylated CpGs with *p*-value < 0.0001 for functional enrichment with GO terms and KEGG pathways^56^ via the gometh function in the *missMethyl* package in R/Bioconductor^100^.

### Replication study for DMPs in an independent cohort

Eleven mother-infant pairs from a Taiwanese cohort had both maternal urine samples and cord blood samples. Details of the study population have been published elsewhere^101^. Briefly, women were recruited from an obstetrics clinic in Cathay General Hospital (CGH) in Taipei, Taiwan from March to December 2010. The eligibility criteria included an age of 18–45 years, <13 weeks pregnant with detection of the fetal heartbeat at the first prenatal visit, and planning to deliver at CGH. The concentrations of BPA in urine samples from pregnant women during the third trimester were measured using ultra-performance liquid chromatography coupled with time-of-flight mass spectrometry as described by Huang *et al*.^77^. The characteristics of the eleven mother-infant pairs are presented in Supplementary Table S10. Genome-wide DNA methylation was assessed as follows: cord blood DNA (500 ng) was subjected to bisulfite conversion using a Zymo EZ DNA Methylation Kit (Zymo Research, Irvine, CA, USA). Following bisulfite conversion, DNA was hybridized to the Infinium HumanMethylation 450 BeadChip (Illumina Inc.) and scanned according to the manufacturer’s protocol. Raw data were processed with the Chip Analysis Methylation Pipeline Bioconductor package (v1.8.0) in the R environment. Probes meeting any of the following conditions were removed: (1) internal controls, (2) detected *p*-value < 0.01, (3) <3 beads in at least 5% of the samples per probe, (4) aligned to multiple locations, (5) contained SNPs, or (6) were located on sex chromosomes. A total of 433,523 probes passed the filtering process, and intra-array normalization was performed with the BMIQ (Beta MIxture Quantile dilation) method. The ComBat method^96^ was applied to reduce the batch effect. Finally, β-values batch-corrected were obtained. Linear regression analyses adjusted for the sex of child were used to determine the associations of the β-value with *ln*-transformed BPA levels. We assumed that we could compare the direction of methylation changes related to BPA levels between two cohorts using β-values batch-corrected by the ComBat even though the normalization methods were different. Due to the minimal sample size (n = 11), we did not stratify the analysis by sex.

### Ethics

Written informed consents were obtained from all participants. The institutional Ethical Board for human gene and genome studies at the Hokkaido University Graduate School of Medicine and the Hokkaido University Center for Environmental and Health Science approved the study protocol. The study protocol of Taiwan cohort was approved by the Institutional Review Board of Cathay General Hospital (CGH) in Taipei, Taiwan. All experiments were performed in accordance with relevant guidelines and regulations.

## Supplementary information

Dataset 1

Detaset 2
