# Supplementary material for: An epigenome-wide analysis of cord blood DNA methylation reveals sex-specific effect of exposure to bisphenol A
Source: Sci Rep. 2019 Aug 26;9:12369. doi: 10.1038/s41598-019-48916-5 (PMC6710292; doi:10.1038/s41598-019-48916-5)
Supplement: Supplementary file 1 — Dataset 1 [file 41598_2019_48916_MOESM1_ESM.docx]

**Supplementary Information**

**An epigenome-wide analysis of cord blood DNA methylations reveals sex-specific effect of exposure to bisphenol A**

Ryu Miura^1^, Atsuko Araki^1^, Machiko Minatoya^1^, Kunio Miyake^2^, Mei-Lien Chen^3^, Sumitaka Kobayashi^1^, Chihiro Miyashita^1^, Jun Yamamoto^4^, Toru Matsumura^4^, Mayumi Ishizuka^5^, Takeo Kubota^6^, Reiko Kishi^1,*^

^1^Hokkaido University Center for Environmental and Health Sciences, Sapporo, Japan

^2^Department of Health Sciences, Interdisciplinary Graduate School of Medicine and Engineering, University of Yamanashi, Japan

^3^Institute of Environmental and Occupational Health Sciences, National Yang Ming University, Taipei, Taiwan

^4^Institute of Environmental Ecology, Idea Consultants, Inc., Shizuoka, Japan

^5^Department of Environmental Veterinary Sciences, Graduate School of Veterinary Medicine, Hokkaido University, Sapporo, Japan

^6^Faculty of Child Studies, Seitoku University, Chiba, Japan

**Supplementary Table S1.** Comparison of BPA levels and maternal and infant characteristics between infant’s sexes.

|  |  |  | Male infants | Female infants | *p*-value^d^ |
| --- | --- | --- | --- | --- | --- |
|  |  |  | (n=123) | (n=154) |  |
|  |  |  | N (%) or Mean ± SD | |  |
| **BPA concentration in cord blood** | | | | | |
|  | Continuous (ng/mL)^a^ | | 0.056 ± 0.03 | 0.056 ± 0.04 | 0.429 |
|  | Below LOQ^c^ | | 37 (30.1) | 50 (32.5) | 0.671 |
| **Maternal characteristic** | | | | | |
|  | Maternal age (year)^b^ | | 30.1 ± 5.0 | 30.0 ± 4.9 | 0.852 |
|  | Prenatal-BMI (kg/m^2^)^b^ | | 20.8 ± 2.8 | 21.0 ± 3.0 | 0.514 |
|  | Parity^c^ | 0 | 67 (54.5) | 78 (50.6) | 0.527 |
|  |  | ≧ 1 | 56 (45.5) | 76 (49.4) |  |
|  | Educational level (year)^c^ | | | | |
|  |  | ≦ 12 | 48 (39.0) | 75 (48.7) | 0.107 |
|  |  | > 12 | 75 (61.0) | 79 (51.3) |  |
|  | Annual household income (million yen)^c^ | | | | |
|  |  | < 3 | 20 (16.3) | 31 (20.4) | 0.641 |
|  |  | 3-5 | 69 (56.1) | 75 (49.3) |  |
|  |  | 5-7 | 24 (19.5) | 35 (23.0) |  |
|  |  | > 7 | 10 (8.1) | 11 (7.2) |  |
|  | Smoking during pregnancy^c^ | | | | |
|  |  | No | 107 (87.0) | 127 (82.5) | 0.302 |
|  |  | Yes | 16 (13.0) | 27 (17.5) |  |
|  | Alcohol consumption during pregnancy^c^ | | | | |
|  |  | No | 81 (65.9) | 102 (66.2) | 0.947 |
|  |  | Yes | 42 (34.1) | 52 (33.8) |  |
|  | Caffeine intake during pregnancy (mg/day)^a^ | | | | |
|  |  |  | 152.0 ± 109.7 | 146.0 ± 131.3 | 0.489 |
|  |  |  |  |  |  |
| **Infant characteristics** | | | | | |
|  | Gestational age (weeks)^b^ | | 39.7 ± 0.9 | 39.8 ± 1.1 | 0.627 |
|  | Birth weight (g)^b^ | | 3200.7 ± 307.8 | 3075.9 ± 343.6 | 0.002 |

^a^Mann-Whitney U-test, ^b^Independent t-test, ^c^Chi- squared test

^d^*p*-value between males and females

**Supplementary Figure S1**. Quantile-quantile plots of the log_10_(*p*-values) for the epigenome-wide analysis of the associations between BPA exposure and DNA methylation in cord blood among all newborns (A), male infants (B), and female infants (C).

^a^Adjusted for maternal age, maternal educational levels, maternal pre-pregnancy BMI, maternal smoking during pregnancy, gestational age, infant sex, and cord blood cell estimates.

^b^Adjusted for maternal age, maternal educational levels, maternal pre-pregnancy BMI, maternal smoking during pregnancy, gestational age, cord blood cell estimates.

**Supplementary Table S2.** CpGs with *p*-value < 0.0001 in the analysis for all newborns.

^f^overlapped with CpGs among females

^m^overlapped with CpGs among males

[EXCEL file]

**Supplementary Table S3.** CpGs with *p*-value < 0.0001 in the analysis for male infants.

[EXCEL file]

**Supplementary Table S4.** CpGs with *p*-value < 0.0001 in the analysis for female infants.

[EXCEL file]

**Supplementary Figure S2.** Common significant CpGs found in analyses for all newborns and male or female infants (selected based on *p* < 0.0001).

**Supplementary Figure S3.** The percentage of hypomethylated and hypermethylated CpGs with *p*-value < 0.0001 found in the sex-stratified analyses (A) excluding infants with sustained maternal smoking during pregnancy (male: n=16, female: n=27), (B) excluding infants with BPA value below LOQ (male: n=37, female: n=50), and (C) for the associations between DNA methylation and tertile BPA levels (< 0.041, 0.041−0.066, and > 0.066 ng/mL).

**Supplementary Table S5.** CpGs with *p*-value < 0.0001 in the analysis of the associations between DNA methylation and tertile BPA levels among all newborns.

[EXCEL file]

**Supplementary Table S6.** Supplemental Table S6. CpGs with *p*-value < 0.0001 in the analysis of the associations between DNA methylation and tertile BPA levels among male infants.

*male-specific DMPs as shown in Table 2.

[EXCEL file]

**Supplementary Table S7.** Supplemental Table S7. CpGs with *p*-value < 0.0001 in the analysis of the associations between DNA methylation and tertile BPA levels among female infants.

*female-specific DMPs as shown in Table 2.

[EXCEL file]

**Supplementary Figure S4.** Distribution of BPA levels in cord blood. For 87 samples below the limit of quantification (LOQ, 0.04 ng/mL), we assigned a value of half the detection limit (0.02 ng/mL). Median (25^th^ to 75^th^ percentiles) of BPA concentrations in cord blood was 0.05 ng/mL (IQR: < LOQ – 0.07).

**Supplementary Table S8.** Correlation of cord blood cell estimates based on 450K methylation array profiles with BPA levels among all infants, infants with < LOQ, and infants with > LOQ.

|  | All infants (n=277) | | |  | < LOQ (n=87) | | |  | > LOQ (n=190) | | |
| --- | --- | --- | --- | --- | --- | --- | --- | --- | --- | --- | --- |
|  | Correlation  (ρ) | | *p*-value |  | Correlation  (ρ) | | *p*-value |  | Correlation  (ρ) | | *p*-value |
|  |  |  |  |  |  |  |  |  |  |  |  |
| CD8T | -0.062 |  | 0.303 |  | -0.017 |  | 0.854 |  | -0.089 |  | 0.272 |
| CD4T | -0.089 |  | 0.140 |  | -0.106 |  | 0.242 |  | -0.067 |  | 0.412 |
| NK cells | 0.031 |  | 0.605 |  | 0.081 |  | 0.373 |  | -0.012 |  | 0.882 |
| B cells | -0.038 |  | 0.530 |  | -0.031 |  | 0.731 |  | -0.025 |  | 0.761 |
| Monocytes | 0.116 |  | 0.055 |  | 0.147 |  | 0.106 |  | 0.107 |  | 0.188 |
| Granulocytes | 0.053 |  | 0.376 |  | 0.004 |  | 0.968 |  | 0.075 |  | 0.359 |
| nRBCs | -0.044 |  | 0.471 |  | -0.006 |  | 0.944 |  | -0.075 |  | 0.358 |

ρ, Spearman's correlation test

**Supplementary Table S9.** Median and Interquartile Range (IQR) of cord blood cell estimates among all infants, males, and females.

|  | Median (IQR) | | | | | *p*-value^a^ |
| --- | --- | --- | --- | --- | --- | --- |
|  | All infants |  | Males |  | Females |  |
| CD8T | 0.11 (0.09-0.14) |  | 0.12 (0.10-0.15) |  | 0.11 (0.09-0.13) | 0.03 |
| CD4T | 0.14 (0.11-0.17) |  | 0.14 (0.11-0.17) |  | 0.14 (0.11-0.17) | 0.80 |
| NK cells | 0.00 (0.00-0.02) |  | 0.02 (0.00-0.04) |  | 0.02 (0.00-0.03) | 0.72 |
| B cells | 0.08 (0.07-0.10) |  | 0.08 (0.07-0.10) |  | 0.09 (0.07-0.10) | 0.79 |
| Monocytes | 0.08 (0.06-0.09) |  | 0.07 (0.06-0.09) |  | 0.08 (0.07-0.09) | 0.42 |
| Granulocytes | 0.54 (0.48-059) |  | 0.53 (0.48-0.59) |  | 0.54 (0.48-0.60) | 0.28 |
| nRBCs | 0.07 (0.05-0.09) |  | 0.07 (0.06-0.09) |  | 0.07 (0.05-0.08) | 0.08 |

^a^*p*-value between males and females was obtained by Mann-Whitney’s test.

**Supplementary Table S10.** Characteristics of subjects from the Taiwanese cohort (n=11).

|  |  |  |  | N (%) or Mean ± SD | |
| --- | --- | --- | --- | --- | --- |
|  |  |  |  |  |  |
| **Creatinine-adjusted BPA concentration in maternal urine (μg/g creatinine)** | | | | 23.5 ± 48.0 | |
|  |  |  |  |  |  |
|  |  |  |  |  |  |
| **Maternal characteristics** | | | | |  |
|  | Maternal age (years) | |  | 30.2 ± 4.0 | |
|  | Prenatal-BMI (kg/m^2^) | |  | 21.0 ± 4.0 | |
|  | Educational level (year) | | | |  |
|  |  | ≦ 12 |  | 1 (9.1) | |
|  |  | > 12 |  | 10 (90.9) | |
|  | Smoking during pregnancy | | | |  |
|  |  | No |  | 11 (100) | |
|  |  | Yes |  | 0 (0) | |
|  |  |  |  |  | |
| **Infant characteristics** | | | | |  |
|  | Gestational age (weeks) | |  | 38 ± 1.5 | |
|  | Sex | Male |  | 5 (45.5) | |
|  |  | Female |  | 6 (54.5) | |
|  | Birth weight (g) | |  | 2817.3 ± 263.6 | |
